# Neurosyphilis in Africa: A systematic review

**DOI:** 10.1371/journal.pntd.0005880

**Published:** 2017-08-31

**Authors:** Michael Marks, Joseph N. Jarvis, William Howlett, David C. W. Mabey

**Affiliations:** 1 Clinical Research Department, Faculty of Infectious and Tropical Diseases, London School of Hygiene & Tropical Medicine, London, United Kingdom; 2 Hospital for Tropical Diseases, Mortimer Market Centre, London, United Kingdom; 3 Botswana Harvard AIDS Institute Partnership, Gaborone, Botswana; 4 Division of Infectious Diseases, Perelman School of Medicine, University of Pennsylvania, United States of America; 5 Kilimanjaro Christian Medical Centre, Moshi, Tanzania; University of Tennessee, UNITED STATES

## Abstract

**Introduction:**

Neurological involvement is one of the most important clinical manifestations of syphilis and neurological disease occurs in both early and late syphilis. The impact of HIV co-infection on clinical neurosyphilis remains unclear. The highest prevalence of both syphilis and HIV is in Africa. Therefore it might be expected that neurosyphilis would be an important and not uncommon manifestation of syphilis in Africa and frequently occur in association with HIV co-infection; yet few data are available on neurosyphilis in Africa. The aim of this study is to review data on neurosyphilis in Africa since the onset of the HIV epidemic.

**Methods:**

We searched the literature for references on neurosyphilis in Africa for studies published between the 1^st^ of January 1990 and 15^th^ February 2017. We included case reports, case series, and retrospective and prospective cohort and case-control studies. We did not limit inclusion based on the diagnostic criteria used for neurosyphilis. For retrospective and prospective cohorts, we calculated the proportion of study participants who were diagnosed with neurosyphilis according to the individual study criteria. Depending on the study, we assessed the proportion of patients with syphilis found to have neurosyphilis, and the proportion of patients with neurological syndromes who had neurosyphilis. Due to heterogeneity of data no formal pooling of the data or meta-analysis was undertaken.

**Results:**

Amongst patients presenting with a neurological syndrome, three studies of patients with meningitis were identified; neurosyphilis was consistently reported to cause approximately 3% of all cases. Three studies on stroke reported mixed findings but were limited due to the small number of patients undergoing CSF examination, whilst neurosyphilis continued to be reported as a common cause of dementia in studies from North Africa. Ten studies reported on cases of neurosyphilis amongst patients known to have syphilis. Studies from both North and Southern Africa continue to report cases of late stage syphilis, including tabes dorsalis and neurosyphilis, in association with ocular disease.

**Discussion:**

This is the first systematic review of the literature on neurosyphilis in Africa since the beginning of the HIV epidemic. Neurosyphilis continues to be reported as a manifestation of both early and late syphilis, but the methodological quality of the majority of the included studies was poor. Future well-designed prospective studies are needed to better delineate the incidence and clinical spectrum of neurosyphilis in Africa and to better define interactions with HIV in this setting.

## Author summary

Involvement of the central nervous system is an important manifestation of syphilis which may be more common in patients co-infected with HIV. As most cases of syphilis and HIV are seen in Africa it might be anticipated that neurosyphilis was common there. We reviewed all published material on neurosyphilis in Africa since 1990. There were few well designed studies of neurosyphilis. A small number of studies suggested that syphilis remains a cause of meningitis in Africa. Our data suggest that neuosyphilis remains an important disease in Africa but better prospective studies are needed to understand its epidemiology.

## Introduction

Syphilis, caused by *Treponema pallidum* subsp. *pallidum*, remains an important sexually transmitted disease worldwide. Some studies suggest the natural history, outcomes of treatment, and likelihood of central nervous system involvement are different in HIV-infected compared to uninfected individuals [1–3], but consensus has not been reached [4]. Globally the highest rates of syphilis and HIV are in Africa[5]. Based on this and the potential for interaction with HIV in the region, it might be expected that neurosyphilis would be an important and not uncommon manifestation of syphilis in Africa, yet few data are available.

Neurological involvement is one of the most important manifestations of syphilis. Typically neurosyphilis is described as a late manifestation, but neuroinvasion and neurological disease occur in both early and late syphilis[6], and *T*.*pallidum* may be frequently identified in the CSF of patients with early stage syphilis[7]. The clinical spectrum seen ranges from asymptomatic neuroinvasion, meningitis, meningovascular disease presenting as a stroke-like syndrome, and the late stage manifestations of tertiary syphilis: tabes dorsalis and general paresis of the insane[8].

Cerebrospinal fluid (CSF) is frequently abnormal in patients with neurosyphilis with both pleocytosis and raised protein concentration. The Venereal Disease Research Laboratory (VDRL) assay on CSF is normally considered the gold standard for specificity, but is recognised to have limited sensitivity [9,10]. Other CSF tests, including serological assays, such as the Rapid Plasma Reagin (RPR)[11], Fluorescent Treponemal Antibody-adsorption (FTA-ABS)[12] and *Treponema pallidum* haemagglutination assay[13]and molecular assays including PCR[14] have all been assessed for CSF and have variable specificity and sensitivity for the diagnosis of neurosyphilis. Difficulties in interpretation of CSF pleocytosis in individuals co-infected with HIV add to challenges in evaluating the relationship between the two diseases. CSF pleocytosis is seen in individuals with either infection alone [4,14], thus discerning the cause of pleocytosis in co-infected individuals is not possible.

The aim of this study is to review data on neurosyphilis in Africa since the onset of the HIV epidemic.

## Methods

### Search strategy and selection criteria

We searched Pubmed, Medline, EMBASE and the grey literature for references on neurosyphilis in Africa. We searched reference lists of selected papers to identify additional references. We searched for (“CSF” OR “lumbar puncture” OR “meningitis” OR “meningovascular” OR “stroke”) AND “syphilis”) OR (“neurosyphilis” OR “tabes dorsalis” OR “general paresis”) AND (Africa OR each individual country in Africa). We limited the search to studies published between 1^st^ of January 1990 and 15^th^ February 2017 (the date the search was conducted). No language restrictions were placed. We excluded reviews if they did not report new primary material, studies limited purely to comparisons of diagnostic techniques, studies on non-neurological manifestations of syphilis, studies reporting cases occurring before 1990, and studies reporting patients already described in a different paper.

### Data extraction

For each reference we extracted the number of sites in each study, the duration of the study, inclusion and exclusion criteria, diagnostic criteria for neurosyphilis, the clinical syndromes, and the number of HIV infected and uninfected individuals. We also extracted information on the treatment and outcomes of patients in these studies.

### Statistical analysis

For retrospective and prospective cohorts, we calculated the proportion of study participants who were diagnosed with neurosyphilis according to the individual study criteria. Depending on the study, we assessed the proportion of patients with syphilis found to have neurosyphilis, and the proportion of patients with neurological syndromes who had neurosyphilis. Due to heterogeneity of data no formal pooling of the data or meta-analysis was undertaken.

## Results

**Fig 1:**
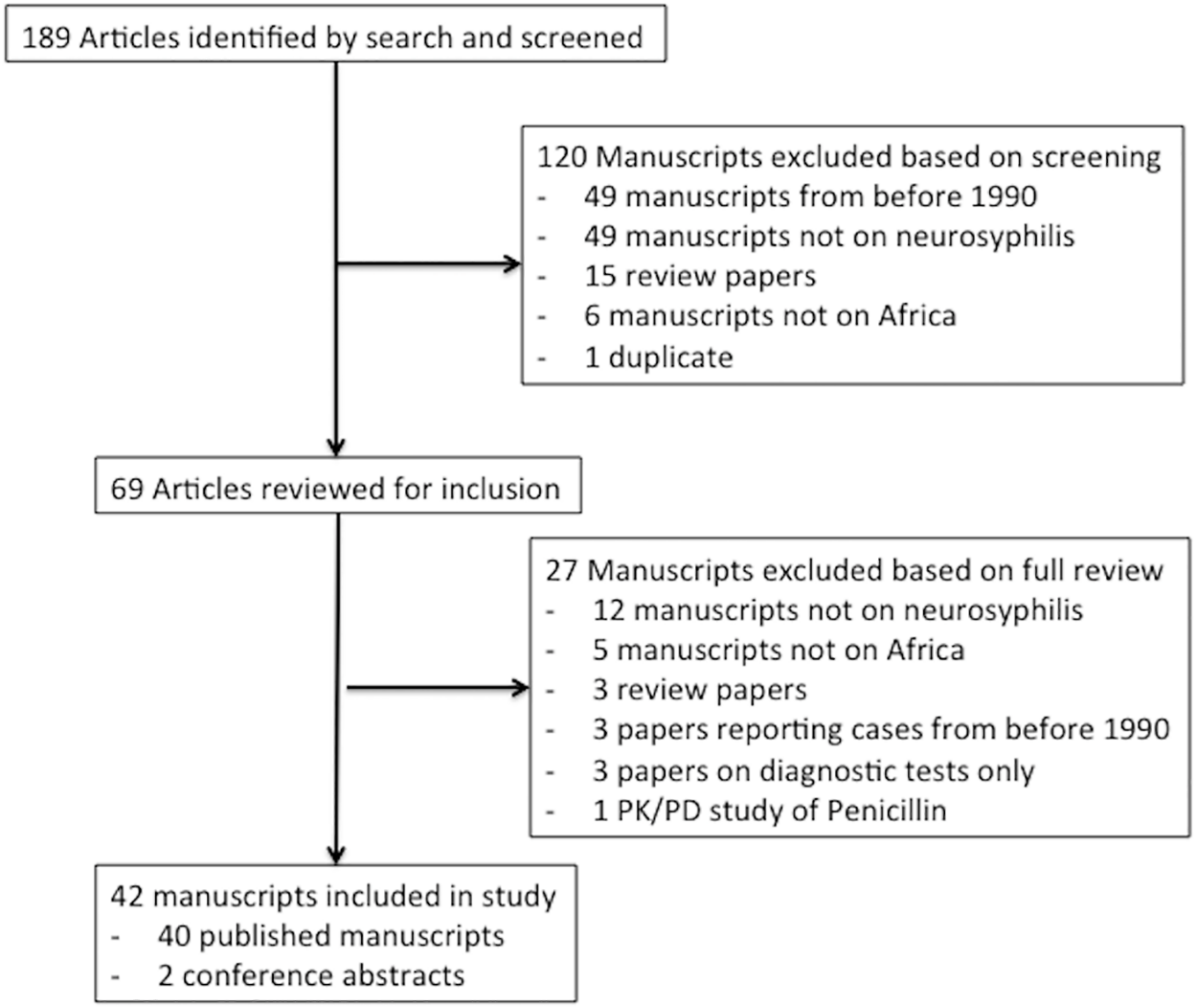
Study inclusion flowchart.

We identified 189 citations of which 42 met our inclusion criteria (Fig 1) (Tables 1–3).

**Table 1:** Case reports of neurosyphilis—Clinical and diagnostic features.

Study	Country	n	Year	CSF Criteria for Diagnosis of Neurosyphilis	Clinical Syndrome	% HIV Positive Patients
Wolf B[15]	Zimbabwe	2	1993	RPR / TPHA	One case of early meningitic disease & one case of paranchymatous disease	0%
Silber E—1998[16]	South Africa	7	1998	VDRL	Meningitis	14% (n = 1)
Bulundwe KK[17]	South Africa	1	2000	VDRL	Meningitis	100%
Nunes da Silva MJ[18]	Guinea-Bissau	1	2000	VDRL / TPHA	Meningitis	0%
Seydi M[19]	Senegal	1	2001	VDRL / TPHA	Meningitis	100%
Corr P[20]	South Africa	1	2004	VDRL / FTA	Meningovascular	100%
Zouhair K[21]	Morocco	4	2004	Not Stated	Tabes Dorsalis	0%
Harifi G[22]	Morocco	1	2009	TPHA / VDRL	Trigeminal Neuralgia	Not Stated
Salami AK[23]	Nigeria	1	2009	VDRL	Meningovascular	100%
Mrabet D[24]	Tunisia	1	2010	VDRL	Tabes Dorsalis	0%
Kyebambe PS[25]	Uganda	1	2010	VDRL	Meningovascular	100%
Wasserman S[26]	South Africa	1	2011	VDRL	Guillan-Barre Syndrome	0%
Mebrouk Y[27]	Morocco	1	2011	VDRL / TPHA	Spinal Meningitis	0%
Napon C[28]	Burkina Faso	1	2012	VDRL / TPHA	Focal Neurology	0%
Derouich I[29]	Morocco	1	2013	VDRL / TPHA	Limbic Encephalitis	0%
Samia M[30]	Morocco	1	2013	Syphilis serology not otherwise specified	Tabes Dorsalis	0%
Lakjiri S[31]	Morocco	1	2014	Syphilis serology not otherwise specified	Tabes Dorsalis	0%
Moolla Y[32]	South Africa	1	2016	VDRL	Psychosis	0%
Hajjaj I[33]	Morocco	1	2010	TPHA / VDRL	Meningitis	0%
Bourazza A[34]	Morocco	5	2008	VDRL	Meningovascular	0%
Hsaini Y[35]	Morocco	3	2010	Syphilis serology not otherwise specified	Tabes Dorsalis	0%

**Table 2:** Patients presenting with neurological syndromes—Inclusion criteria, clinical and diagnostic features.

Study	Country	n	Year	Study Design	Study Duration	Study Inclusion Criteria	CSF Criteria for Diagnosis of Neurosyphilis	Neurosyphilis Syndrome	Prevalence of Neurosyphilis	% HIV Positive Patients
Benabdeljili M[36]	Morocco	563	2016	Retrospective	13 years	Patients with dementia between 2000–2013	Not Stated	General Paresis of the Insane	3.60%	Not Stated
Amare A[37]	Ethiopia	25 tested for neurosyphilis (total study sample size n = 119)	2008	Retrospective	10 years	Status Epilepticus in patients aged ≥13	VDRL	Status Epilepticus	8%	Not stated
Siddiqi OK[38]	Zambia	81	2015	Prospective	23 months	New Seizure in HIV Adults	VDRL		0%^#^	100%
Chraa M[39]	Morocco	128	2014	Retrospecive	4 Years	Stroke in adults 18–45	Not Stated	Meningovascular Syphilis	7.80%	Not Stated
Kumwenda JJ[40]	Malawi	59	2005	Prospective	8 months	Presentation with Stroke	VDRL	Meningovascular Syphilis	0%	0.48
Sokrab TE[41]	Sudan	96	2002	Prospetive	Not Given	Presentation with Stroke	VDRL	Meningovascular Syphilis	4.20%	Not Stated
de Mast Q[42]	Tanzania	158 cases 369 controls	2016	Prosepctive Case Conrol Study	3 Years	Case Control Study: Presentation with Stroke	No CSF Testing Treponemal IgG in Blood	Meningovascular Syphilis	^N/A^	24.5% in cases 6.5% in controls
Silber E—1999[43]	South Africa	60	1999	Prospective	5 months	Suspected Meningitis	RPR / TPHA	Meningitis	3.30%	68.00%
Jarvis J[44]	South Africa	4,961	2010	Retrospective	3 years	Suspected Meningitis	TPHA / VDRL	Meningitis	1.27%^	XX
Rajasingham R[45]	Uganda	117	2015	Prospective	2 years	HIV with suspected meningitis	VDRL	Meningitis	2.70%	100%
Szabo I[46]	Uganda and Kenya	288	2013	Prospecive	4 Months	Patients presenting with neurology—not further specified	Not Stated		11.1%*	12.15%

^ Of the 4,961 individuals undergoing lumbar puncture for suspected meningitis 2,291 were found to have normal CSF and a further 931 patients were found to have changes compatible with HIV alone. 1,737 individuals had an LP compatible with meningitis of whom 22 were diagnosed with neurosyphilis.

* The diagnosis explaining the patients neurology is stated to be Syphilis in 32 patients in this study but clear CSF diagnostic criteria are not stated

^#^ 5% of patients were found to have postive syphilis serology but were negative on testing of the CSF

**Table 3:** Patients with syphilis investigated for neurosyphilis—Inclusion criteria, clinical and diagnostic features.

Study	Country	n	Year	Study Design	Study Duration	Study Inclusion Criteria	CSF Criteria for Diagnosis of Neurosyphilis	Neurosyphilis Syndrome	Prevalence of Neurosyphilis	% HIV Positive Patients
Yahyaoui M[47]	Morroco	201	2005	Retrospective	10 years	Clinical diagnosis of Neurosyphilis with positive CSF serology	VDRL or TPHA	Dementia n = 145 Meningovascular n = 45 Tabes dorsalis n = 20 Ocular Syphilis n = 13 Other n = 21	100%	29 Patients tested with prevalence 6.9%
Rafai M.A [48]	Morocco	55	2012	Retrospective	12 Years	Clinical diagnosis of Neurosyphilis with positive CSF serology	Syphilis Serology not otherwise specified	General Paresis of the Insane n = 24 Meningovascular Syphilis n = 20 Tabes Dorsalis n = 9 Other n = 3	100%	0%
Allali F[49]	Morocco	24	2006	Retrospective	20 years	Arthropathy due to Tabes Dorsalis	Treponemal Antibodies	Tabes Dorsalis	33.30%	0%
Timmermans M[50]	South Africa	161	2004	Retrospective	10 years	Positive CSF FTA	CSF FTA	Dementia n = 82 Stroke n = 24 Spinal cord disease n = 15 Seizures n = 14	100%	0%
Molepo J[51]	South Africa	50	2007	Prospective	15 Months	Suspected Neurosyphilis	VDRL / FTA		80%^	Not Stated
Cisse A—2002[52]	Guinea	28	2002	Retrospective	8 Years	Neurosyphilis with focal neurology	VDRL / TPHA	Focal neurology*	100%	0%
Reekie I[53]	South Africa	31	2016	Retrospective	20 months	Patients with clinical Ocular Syphilis	CSF Pleocytosis / FTA	Ocular Syphilis	25.80%	50% of patients with Neurosyphilis
Tattevin P[54]	Mozambique	21	2002	Prospective	20 Months	Latent Syphilis in HIV positive patients	Protein >50mg/dl WBC > 5/mm Positive CSF RPR test	Asymptomatic Neurosyphilis	19%	100%
Nnoruka EN[55]	Nigeria	31	2005	Prospective	8 Months	Patients with HIV & Syphilis seen in Dermatology Clinic	VDRL	Asymptomatic Neurosyphilis	0%	100%
Modi G[56]	South Africa	506	2007	Prospective	6 Months	All HIV Positive Patients	Syphilis Serology not otherwise specified	Asymptomatic Neurosyphilis	0.20%	100%

^ A further 10% of patients negative by serology were reported to be PCR positive for T.pallidum in CSF

* The study authors report that as well as these 'atypical cases' they 11 cases of Tabes dorsalis, 4 cases of syphilis meningitis, 8 cases of meningovascular syphilis and 13 cases of general paresis of the insane

### Case reports

Twenty-one case reports including 37 patients were identified [15–35]. The clinical syndromes included focal neurological manifestations such as cranial nerve involvement and including range of manifestations including meningitis, meningovascular syphilis, tabes dorsalis and dementia (Table 1).

### Patients presenting with a neurological syndrome

Eleven studies reported data on patients presenting with neurological syndromes (Table 2). The methodological quality of many of these studies was poor (Table 4). At least one major methodological limitation was identified in half. Six studies were retrospective (54.5%) and only eight (72.7%) included the performance of lumbar punctures to make the diagnosis of neurosyphilis. Several studies did not provide clear diagnostic criteria for neurosyphilis and, even in studies where LP was performed, it was frequently not performed on all patients enrolled in the study.

**Table 4:** Methodological assessment of criteria used for diagnosis of neurosyphilis.

Study	Study Methodology	Criteria for diagnosis of Neurosyphilis			
		LP required for diagnosis	LP performed on all patients	Testing for Neurosyphilis Performed on all CSF samples	CSF Diagnostic Criteria Stated
Benabdeljili M[36]	Retrospective	Yes	Yes	Yes	Not Clearly Defined
Amare A[37]	Retrospective	Not Stated	No	No	Clearly Defined
Siddiqi OK[38]	Prospective	Yes	Yes	Yes	Clearly Defined
Chraa M[39]	Retrospective	No	Testing Not Peformed	Testing Not Peformed	Not Performed
Kumwenda JJ[40]	Prospective	Yes	No	Yes	Clearly Defined
Sokrab TE[41]	Prospective	Not Stated	Not Stated	Not Stated	Clearly Defined
de Mast Q[42]	Prosepctive Case Conrol Study	No	Testing Not Peformed	Testing Not Peformed	Not Performed
Silber E—1999[43]	Prospective	Yes	Yes	Yes	Clearly Defined
Jarvis J[44]	Retrospective	Yes	Yes	No	Clearly Defined
Rajasingham R[45]	Prospective	Yes	Yes	Yes	Clearly Defined
Szabo I[46]	Prospecive	Not stated	No	Not stated	Not Clearly Defined
Yahyaoui M[47]	Retrospective	Yes	Yes	Yes	Clearly Defined
Rafai M.A [48]	Retrospective	Yes	Yes	Yes	Not Clearly Defined
Allali F[49]	Retrospective	Yes	Yes	Yes	Not Clearly Defined
Timmermans M[50]	Retrospective	Yes	Yes	Yes	Clearly Defined
Molepo J[51]	Prospective	Yes	Yes	Yes	Clearly Defined
Cisse A—2002[52]	Retrospective	Yes	Yes	Yes	Clearly Defined
Reekie I[53]	Retrospective	Yes	No	No	Clearly Defined
Tattevin P[54]	Prospective	Yes	Yes	Yes	Clearly Defined
Nnoruka EN[55]	Prospective	Not Stated	Not Stated	Not Stated	Clearly Defined
Modi G[56]	Prospective	Yes	Not Stated	Not Stated	Not Clearly Defined

#### Dementia

In a single-centre retrospective study from Morocco of 563 patients attending a dementia clinic between 2000–2013 neurosyphilis was reported to be the most common infectious cause of dementia identified, and accounted for 3.6% of all cases of dementia seen during the study period[36]. Details on CSF diagnostics were not provided, so the proportion of these cases which were definitive neurosyphilis is not known.

#### Seizures

A retrospective study in Ethiopia of individuals aged over 13 presenting with status epilepticus enrolled a total of 119 patients but only 25 (21%) patients underwent CSF testing for neurosyphilis[37]. In this subgroup a diagnosis of neurosyphilis was made in 2 patients on the basis of a positive CSF VDRL (8%).

In a prospective study in Zambia[38], HIV infected patients with a new onset of seizures were enrolled and underwent CSF testing within two weeks of the index seizure. Three patients had a reactive serum RPR and TPHA, but no individual had a positive CSF VDRL.

#### Stroke

In a retrospective study from Morocco of adults aged 18–45 (n = 128) presenting with stroke the underlying diagnosis was reported to be meningovascular syphilis in 14 patients (11%). However CSF diagnostics were not undertaken and syphilis was diagnosed on the basis of serum serological testing, so the proportion of these cases which were definitive neurosyphilis is unknown. [39].

Two prospective studies conducted in Malawi and Sudan enrolled patients presenting with stroke (n = 98 and n = 96 respectively)[40,41]. Lumbar punctures were performed and a positive CSF VDRL was considered diagnostic in both studies. In the study in Malawi [40] 48% of patients were HIV positive. Fifty nine individuals had serum syphilis serology performed. Thirteen patients had a positive TPPA and a negative VDRL and a further five patients had both a positive TPPA and a VDRL. CSF VDRL testing was negative in all patients with positive serological tests on blood. In the study in Sudan both serological and CSF evidence of syphilis were assessed and a positive CSF-VDRL was considered diagnostic of neurosyphilis [41]. The prevalence of HIV was not stated for patients enrolled in the study. Syphilis was identified as the major risk factor for stroke in four patients (4.2%) but no details were given as to whether this was based on serum testing alone or on CSF testing.

A case-control study of stroke conducted in Tanzania between 2003–2006 enrolled 158 stroke cases and 369 age- and sex- matched controls[42]. Serology for syphilis was performed using an IgG ELISA (*Treponema pallidum* IgG ELISA; IBL international GmbH, Hamburg, Germany). The seroprevalence was significantly higher in stroke cases (24.7%) compared to controls (12.7%) and a positive ELISA was associated with a significantly increased risk of stroke (OR 2.8, 95% CI 1.6–4.6). No CSF testing was performed in this study so it is not clear whether this association represents causation and, if so, whether the mechanism is meningovascular syphilis.

#### Meningitis

Prospective and retrospective studies in South Africa, and a further prospective study in Uganda, enrolled patients with clinically suspected meningitis. In the prospective South African study, sixty consecutive patients with clinically suspected meningitis were enrolled[43]. The overall seroprevalence for HIV was 68%. Of the 60 enrolled patients a microbiological diagnosis was made in 39 (65%). Meningococcal meningitis accounted for ten cases. Tuberculous meningitis and cryptococcal meningitis each accounted for nine cases. On the basis of a positive CSF RPR and TPHA two cases of neurosyphilis were diagnosed (3.3%)–one with an oculomotor nerve palsy and one with cryptococcal co-infection.

In the retrospective study from South Africa[44], 4,961 episodes of suspected meningitis were included. Neurosyphilis was diagnosed on the basis of a reactive CSF TPHA or VDRL. Of the total population studied, 2,293 were found to have normal CSF and a further 931 patients were found to have changes compatible with HIV alone. Of the 1,737 individuals who had an LP compatible with meningitis a microbiological diagnosis was made in 820 patients (47%). A diagnosis of neurosyphilis was made in 22 patients (1.26% of patients with an abnormal CSF, 3% of patients with a microbiological diagnosis). Testing for neurosyphilis was performed at the physician’s discretion and this is likely to be an underestimate of the true proportion of cases caused by neurosyphilis.

In Uganda samples were obtained from patients enrolled in a study ART initiation in HIV positive patients with cryptococcal meningitis [45]. A total of 63 patients negative for Cryptococcus in CSF and 54 patients with Cryptococcal meningitis were assessed. The overall syphilis seroprevalence in these patients was not stated but neurosyphilis was diagnosed in 3 of 111 (2.7%) patients who were tested on the basis of a positive CSF VDRL, accounting for a comparable number of cases as bacterial meningitis (*Streptococcus pneumoniae* n = 4, *Neisseria meningitidis* n = 1).

#### Other

In a study conducted at a single site in Uganda and a single site in Kenya, a total of 288 consecutive patients with neurological symptoms were enrolled[46]. All patients underwent a neurological examination but detailed information was not provided on the clinical abnormalities seen. The overall prevalence of HIV was 12.2%. A total of 87 patients underwent CSF testing. Neurosyphilis was the diagnosis in 32 (11.1%) although the proportion of patients who had a diagnosis based on a positive CSF test was not reported, nor was the overall prevalence of syphilis in the study population given.

### Studies on neurosyphilis amongst patients with syphilis

Ten studies reported on cases of neurosyphilis amongst patients known to have syphilis (Table 3). The methodological quality of many studies was poor with at least one significant methodological weakness in all but one study (Table 4). The study design was retrospective for six of the studies (60%) whilst lumbar puncture was performed in only seven studies (70%) for the diagnosis of neurosyphilis but, of these, only five studies (50%) stated what assay was used.

#### Suspected neurosyphilis

Two retrospective studies from Morocco reported on a total of 256 patients clinically suspected to have neurosyphilis [47,48]. In the larger study (n = 201), neurosyphilis was diagnosed based on a positive CSF VDRL and TPHA. In the smaller study (n = 55) the authors report patients had a positive CSF serological test for neurosyphilis but do not provide details on which tests were performed. Dementia was the most commonly clinical syndrome seen in 56.6% patients (n = 145) followed by meningovascular syphilis (n = 65, 25.4%) and tabes dorsalis (n = 29, 11.3%).

A further retrospective study from Morocco included 24 patients with arthropathy suspected to be due to tabes dorsalis. CSF testing was performed using a test for treponemal antibodies but the precise test was not stated. In this study all patients had positive syphilis serology on serum while 33.3% of patients had positive CSF serology [49].

A retrospective study from South Africa included patients with neurosyphilis diagnosed on the basis of a positive CSF FTA-ABS seen in a single hospital in the Western Cape region over a ten-year period. A total of 161 patients were identified. Dementia was the most common diagnosis (n = 81, 50.9%), followed by meningovascular syphilis (n = 24, 14.9%)[50].

In a prospective study in South Africa[51], 50 patients with suspected neurosyphilis were enrolled. All patients were seropositive for syphilis and clinically diagnosed with tertiary neurosyphilis although detailed clinical information was not provided. All underwent lumbar puncture. Forty patients (80%) had reactive CSF serology (70% VDRL and FTA-ABS, 10% FTA-ABS alone) and a further 5 patients (10%) had a positive CSF PCR but negative CSF serology.

Finally, a case-series from a single centre in Guinea reported 28 patients who were diagnosed with neurosyphilis on the basis of both a positive CSF VDRL and CSF-TPHA [52]. The series included a broad spectrum of neurological presentations including three cases of facial nerve palsy, four cases of optic atrophy, eleven cases of seizures, three cases of cerebellar ataxia, two cases of vestibular-cochlear nerve dysfunction and five cases of clinical meningitis.

#### Ocular syphilis

A retrospective study from South Africa included patients clinically diagnosed with ocular syphilis (n = 68), all of whom had positive serum serology results. Four cases were known to be HIV positive, one HIV negative, and the status was unknown for the remaining three cases. Thirty-one (45.6%) patients underwent lumbar puncture (not all of whom had CSF serologica testing) and, of these, a quarter (n = 8) had CSF findings felt to be consistent with neurosyphilis [53]. In five cases this was a reactive CSF FTA-ABS and, in three cases, this was a CSF lymphocyic pleocytosis.

#### Individuals with HIV and syphilis co-infection

Three studies were identified reporting on the prevalence of neurosyphilis in individuals with HIV. In a study assessing treatment of HIV co-infected individuals in Mozambique[54], twenty-one patients with latent syphilis underwent lumbar puncture at baseline. Four patients (19%) were diagnosed with asymptomatic neurosyphilis. None had a positive CSF RPR, all had elevated CSF protein and two had a CSF pleocytosis (WBC > 5/mm^3^). As all patients were co-infected with HIV the significance of a pleocystosis greater than 5/mm^3^ is uncertain. All patients were re-examined at 3, 6 and 12 months. At six months follow-up one patient still had an abnormal CSF (elevated protein and pleocyosis) but no patients had an abnormal CSF by twelve months.

In a study conducted between 2000 and 2001, 31 patients with syphilis and HIV co-infection were seen at a dermatology clinic in Nigeria. All had early syphilis (primary syphilis = 9, secondary syphilis = 20 and latent syphilis = 2). The authors report that no cases of neurosyphilis were seen, but details are not provided on what proportion of patients underwent CSF examination[55].

Finally, in a study of 506 consecutive HIV infected patients in Johannesburg only one case of neurosyphilis was identified [56], but neither the prevalence of syphilis nor the number of patients who underwent lumbar puncture is given, so the interpretation of this finding is extremely limited.

### Diagnostic criteria

CSF diagnostic criteria varied across studies (Table 4). The most commonly used criteria were CSF VDRL alone (n = 14 studies) followed by CSF VDRL combined with either a CSF TPHA (n = 9 studies) or FTA-ABS (n = 2 studies). Two studies defined neurosyphilis as reactive CSF FTA-ABS with or without pleocytosis and two studies used the RPR and TPHA assays on CSF. One study defined neurosyphilis as any one of an abnormal CSF protein, white cell count or CSF RPR assay. Ten studies stated that CSF was tested using serological assays but did not clearly state which diagnostic test was used.

## Discussion

This is the first systematic review of the literature on neurosyphilis in Africa. The methodological quality of the majority of the studies was poor and as such limited conclusions can be drawn. The available data are clearly inadequate data to robustly estimate the prevalence of neurosyphilis in Africa or the proportion of cases of important neurological syndromes due to neurosyphilis. The data are also inadequate to address any potential interaction between HIV and syphilis in this setting.

Whilst HIV has been reported in Africa since the 1980s we restricted our search to papers published after 1990. By this point the HIV epidemic was fully established and any impact of HIV on the risk of neurosyphilis might be anticipated o have become apparent. Whilst we used comprehensive search and MESH terms to identify papers it is possible that some studies on oto-syphilis or other forms of neurosyphilis, or studies which did not explicitly mention Africa (or an African country) might have been missed. Despite these limitations, we believe our comprehensive search strategy is likely to have identified nearly all the relevant research papers for this systematic review.

A major methodological failing of many studies was an inadequate approach to diagnostic criteria for neurosyphilis. A number of studies included only serum testing without CSF examination, whilst others did not adequately report the specific CSF assay performed. In part, this may reflect the lack of an adequate gold-standard. There is a clear need to better define standards for use in routine diagnosis in low-resource settings, but also for use in research studies.

The studies on patients presenting with stroke are all limited by methodological weaknesses. Across these studies there appears to be a consistent association between positive serum syphilis serology and stroke but, as only a minority of studies undertook CSF examination, these studies are difficult to interpret, and whether this reflects neurosyphilis is uncertain. It is possible that the association truly reflects meningovascular syphilis, emboli due to cardiovascular syphilis, or confounding factors. Further studies are needed to better delineate the role of syphilis as a cause of stroke in Africa.

Studies from Morocco continue to report late-stage neurosyphilis, but the majority of studies are of low methodological quality. In comparison there appear to be few cases of tertiary neurosyphilis reported from sub-Saharan Africa. Given the weaknesses of most of the studies it is extremely uncertain whether this represents a true difference in the frequency of tertiary neurosyphilis or simply reflects reporting/publication bias or lack of access to medical resources for diagnosis. It has been hypothesised that the ease of access to short courses of penicillin in the community may have altered the natural history of syphilis, resulting in a lower frequency of late stage manifestations, but this would not explain why cases continue to be seen in North Africa but not sub-Saharan Africa.

There was a small number of higher quality prospective studies enrolling patients with suspected meningitis, in which neurosyphilis was identified [43,45]. These studies had clear inclusion criteria, included adequate data on denominators, and clear diagnostic criteria for neurosyphilis. In these studies the prevalence of neurosyphilis was approximately 3%. In these studies the frequency of neurosyphilis was similar to that of *S*.*pneumoniae* as a cause of meningitis, which likely reflects that patients predominantly had subacute meningitis. This would make syphilis an important and treatable cause of meningitis in Africa. Given the limitations of CSF serological assays, it is likely that the true proportion of meningitis due to neurosyphilis is higher.

The clinical epidemiology of neurosyphilis in Africa remains poorly understood and the majority of studies included in this review were of low methodological quality. Inadequate diagnostics remain a major barrier to progress in our understanding of these important diseases. Future well-designed prospective studies are needed to better delineate the incidence and clinical spectrum of neurosyphilis in Africa.

## Supporting information

S1 Checklist PRISMA checklist. (DOC)
